# MYSM1/miR-150/FLT3 inhibits B1a cell proliferation

**DOI:** 10.18632/oncotarget.11738

**Published:** 2016-08-31

**Authors:** Xiao-Xia Jiang, Yu Liu, Hong Li, Yaping Gao, Rong Mu, Jianping Guo, Jing Zhang, Yan-Mei Yang, Fengjun Xiao, Bing Liu, Changyong Wang, Beifen Shen, Si-Yi Chen, Zhanguo Li, Guang Yang

**Affiliations:** ^1^ Beijing Institute of Basic Medical Sciences, Beijing, China; ^2^ Department of Rheumatology and Immunology, People's Hospital, Peking University, Beijing, China; ^3^ Institute of Radiation Medicine, Beijing, China; ^4^ 307-Ivy Translational Medicine Center, Laboratory of Oncology, Affiliated Hospital of Academy of Military Medical Sciences, Beijing, China; ^5^ Department of Molecular Microbiology and Immunology, Norris Comprehensive Cancer Center, Keck School of Medicine, University of Southern California, Los Angeles, CA, USA; ^6^ State Key Laboratory of Toxicology and Medical Countermeasures, Beijing Institute of Pharmacology and Toxicology, Beijing, China

**Keywords:** MYSM1, miR-150, FLT3, B1a, proliferation

## Abstract

The aberrant expansion of B1a cells has been observed in several murine autoimmune disease models; however, the mechanism of such proliferation of B1a cells is still limited. Here, we identify that Myb Like, SWIRM And MPN Domains 1 (MYSM1), a histone H2A deubiquitinase, plays an intrinsic role in the proliferation of B1a cells where MYSM1 deficiency results in the increased proliferation of B1a cells in mice. We demonstrate that MYSM1 recruits c-Myc to the promoter of miR-150 and stimulates the transcription of miR-150. Our further investigation shows that miR-150 decreases FMS-like tyrosine kinase 3 (FLT3) in B1a cells. In agreement with our animal studies, the percentage of FLT3^+^ B1 cells in Systemic Lupus Erythematosus (SLE) patients is significantly higher than healthy control. Thus, this study uncovers a novel pathway MYSM1/miR-150/FLT3 that inhibits proliferation of B1a, which may be involved in the pathogenesis of SLE.

## INTRODUCTION

B1a cells are specialized B cells that constitute only a small fraction of the total B population, but are a significant source of serum antibodies. Additionally, B1a cells make a significant contribution to low-affinity IgM antibodies that are present in the serum of unimmunized mice, known as natural antibodies [1–3]. The natural antibodies of B1a cells play a crucial role in the first-line defense against microbial infections in mice, acting before an adaptive immune response by conventional B2 cells [5–7].

In contrast to conventional B cells (B2 cells), most long-lived B1a cells are thought to be derived from precursors in fetal liver rather than from adult bone marrow. Despite their positive role in first-line antimicrobial defense, elevated B1a cell numbers is often associated with autoimmunity [8]. Some mutant mouse strains with enhanced B cell signaling, such as SHP-1-deficient [11], Lyn-deficient mice [12], or CD22 x Siglec-G double-deficient mice [13], show increased B1a cell numbers and signs of autoimmunity. However, the mechanism of abnormal B1a cell proliferation is far from clear.

MYSM1 is a histone H2A deubiquitinase and its activity in H2A deubiquitination was initially described for the requirement of the activation of several target genes in prostate cancer cells [14]. Our groups as well as other independent groups have observed an essential role of MYSM1 for bone marrow hematopoietic development and lymphocyte generation [15–21]. In spite of these observations, knowledge of the biological functions of MYSM1 remains limited, and its function in B1a cell proliferation had not been investigated.

In the present study, we reveal that MYSM1 plays an important and inhibitory role in B1a cell proliferation. Further mechanistic studies demonstrate that B1a cell proliferation is inhibited by the pathway composed of MYSM1, miR-150, and receptor tyrosine kinase FLT3.

## RESULTS

### Increased frequency of B1a cell population in MYSM1^−/−^ mice

**Figure 1:**
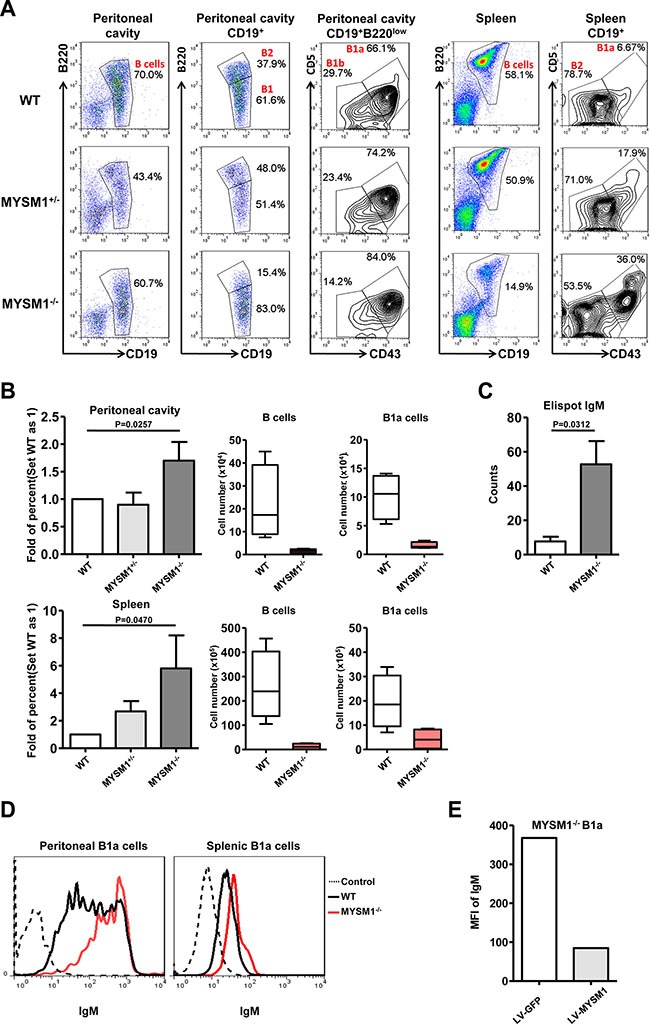
Increased B1a cell frequency and IgM expression in MYSM1 deficient mice (**A**) Representative flow cytometry profiles of B1a cells (CD19^+^B220^lo^CD5^+^CD43^+^) of peritoneal cavity and spleen from homozygous MYSM1^−/−^ mice, MYSM1^+/−^ mice, and WT littermates (*n* = 8 per group, 6 weeks old). Numbers indicate percent of cells in each gate. (**B**) Fold increase of the percentage of MYSM1^−/−^ B1a cells in the peritoneal cavity (top) and spleen (bottom) (set WT as 1) (left), deceased cell number of total B cells (middle) and B1a cells (right) in the peritoneal cavity (top) and spleen (bottom) from MYSM1^−/−^ mice. Data are representative of three independent experiments and shown as the mean ± SD. Significant differences between groups were evaluated using a two-tailed Student's t test. (**C**) IgM production capacity of B1a cells detected by Elispot assay. Data are representative of three independent experiments and shown as the mean ± SD. Significant differences between groups were evaluated using a two-tailed Student's t test. (**D**) Representative flow cytometry profiles of IgM expression in B1a cells of peritoneal cavity and spleen from homozygous MYSM1^−/−^ mice, and WT littermates (*n* = 8 per group, 6 weeks old). (**E**) Rescue assays. Splenic B1a cells from MYSM1^−/−^ mice were stimulated with LPS (100 ng/ml) for 12 hours, then transduced with a control lentiviral vector LV-GFP, or lentiviral vector LV-MYSM1. The mean fluorescent intensity (MFI) of IgM on the B1a cells was detected by flow cytometry after another 48 hours of culture. Data are shown from one of two repeated experiments.

Our previous work demonstrated that MYSM1 deficiency leads to a significant block in the generation of conventional B2 cells [16]. Due to the important role of B1a cells in innate immunity, we further explored the function of MYSM1 in B1a cell development. Cells from the peritoneal cavities and spleens of MYSM1^−/−^ or wild-type (WT) mice were harvested and analyzed by Fluorescence Activated Cell Sorting Cytometry (FACS). B1a cells were defined as CD19^+^B220^lo^CD5^+^CD43^+^ as described by Xiao *et al.* [22] (Figure 1A). As in our previous study [16], our data here show a significantly decreased frequency in total B cells of spleens from MYSM1^−/−^ mice. Unexpectedly, we found that the frequency of B1a cells compared to the total number of B cells from MYSM1^−/−^ mice spleens was increased more than 5-fold when compared with that of WT mice. In the peritoneal cavity, the total B cell frequency within the lymphocyte gate did not show much difference between the MYSM1^−/−^ mice and their WT counterparts, while the proportion of B1a cells to total B cells was increased by a factor of 1.5 (Figure 1A, 1B), though the total number of B1a cells decreased in MYSM1^−/−^ mice. As shown in Supplementary Figure S1, when using CD19^+^CD5^+^ to define B1a cells, we observed similar results with more than a 5-fold increase the proportion of B1a cells in spleens and around 1.5-fold increase the proportion of B1a cells in peritoneal cavities in MYSM1^−/−^ mice compared to WT mice.

B1a cells are a significant source of serum low-affinity polyspecific IgM antibodies. To examine the function of B1a cells in MYSM1^−/−^ mice, CD19^+^B220^lo^CD5^+^CD43^+^ B1a cells were sorted and the production of IgM was detected by Elispot assay. Our data shows that IgM production from B1a cells from MYSM1^−/−^ mice was increased significantly compared to WT mice (Figure 1C, *P* = 0.0312). Furthermore, we examined the expression of surface IgM on B1a cells by FACS analysis, which showed that the expression as determined by the mean fluorescence intensity (MFI) of surface IgM on B1a cells from MYSM1^−/−^ mice was higher than that from WT mice (Figure 1D). To determine if the WT phenotype could be recovered, B cells from the spleens of MYSM1^−/−^ mice were infected with MYSM1-expressing lentivirus (LV-MYSM1), and we found that the expression of surface IgM was dramatically decreased after the level of MYSM1 was recovered in B1a cells from MYSM1^−/−^ mice (Figure 1E).

### MYSM1 stimulates transcription of miR-150 in B1a cells with c-Myc

**Figure 2:**
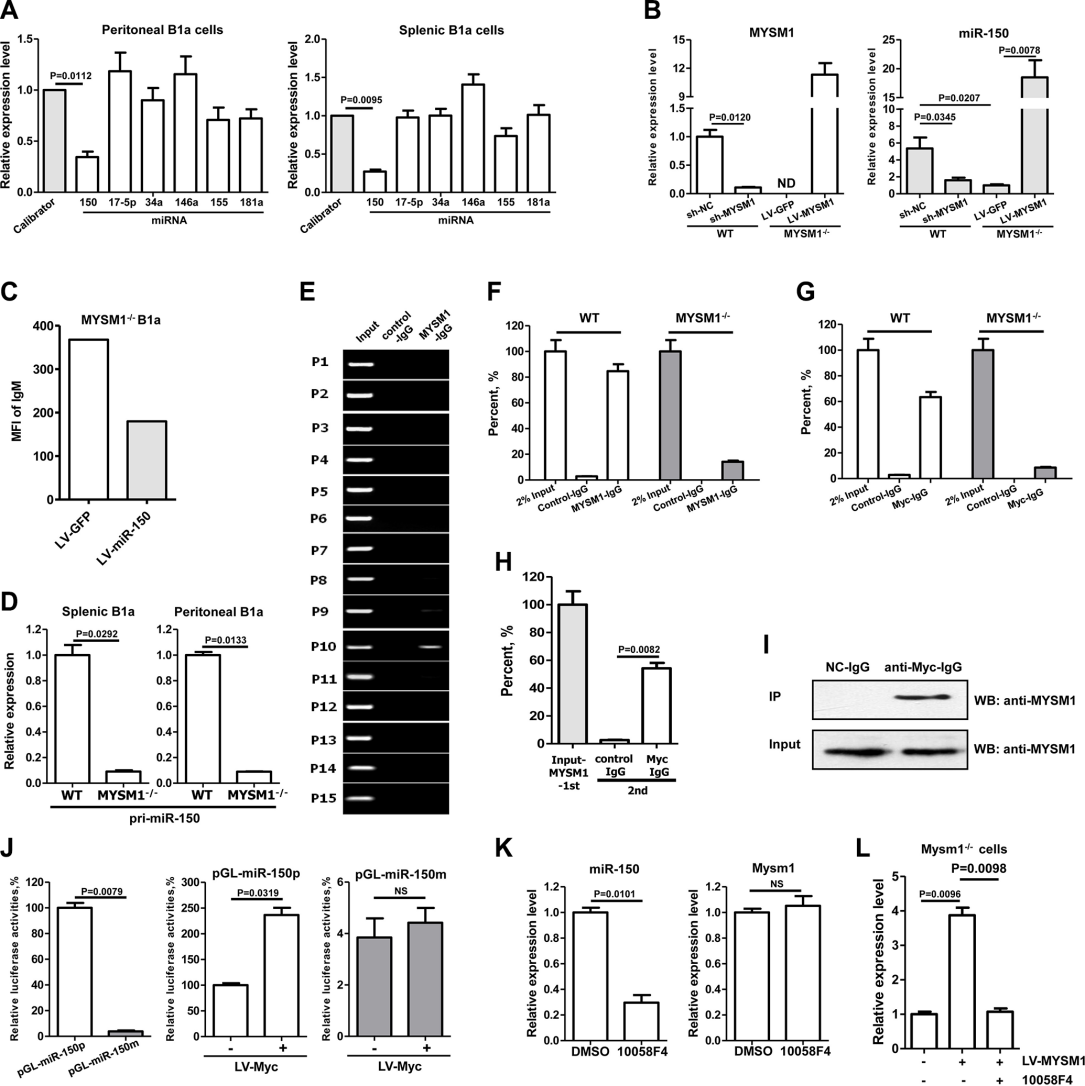
MYSM1 stimulates the transcription of miR-150 in B-1a cells with c-Myc (**A**) miR-150 is decreased in both spleen and peritoneal cavity B1a cells from MYSM1^−/−^ mice. (**B**) The level of miR-150 was decreased after a reduction of MYSM1 (sh-MYSM1) in splenic B1a cells from wild type (WT) mice. Meanwhile, the attenuation of miR-150 in MYSM1^−/−^ splenic B1a cells was reversed with the restoration of MYSM1 expression (LV-MYSM1). (**C**) Surface IgM on B1a cells from MYSM1^−/−^ mice is decreased with elevation of miR-150 (LV-miR-150). (**D**) The levels of pri-miR-150 are significantly reduced in B1a cells from MYSM1^−/−^ mice. (**E**) ChIP assay results show that MYSM1 directly binds to the 1200–1000 nt upstream region of the miR-150 promoter. (**F**, **G**) ChIP assay results show that MYSM1(F) and c-Myc (G) directly binds to the promoter of miR-150 in B1a cells from WT mice, while this interaction could not be detected in B1a cells from MYSM1^−/−^ mice. (**H**) Re-ChIP assay verifies the simultaneous presence of MYSM1 and c-Myc in the promoter of miR-150. (**I**) Co-Immunoprecipitation confirms the interaction between MYSM1 and c-Myc. (**J**) The deletion of -1200∼-1000 nt (pGL-miR-150pm) attenuated luciferase activities of the miR-150-promoter reporter (pGL-miR-150p). In addition, the ectopic expression of c-Myc (LV-Myc) increased the luciferase activities of pGL-miR-150p, but had no effect on pGL-miR-150pm. (**K**) The inhibition of c-Myc attenuates the expression of miR-150. (**L**) Inhibition of c-Myc blocks the elevation of miR-150 induced by restoring MYSM1 (LV-MYSM1). Data are representative of three independent experiments and shown as the mean ± SD. Significant differences between groups were evaluated using a two-tailed Student's *t* test. (NS: Not Significant; ND stands for Not Detectable).

MicroRNAs (miRNAs) are small, non-coding RNAs, containing about 22 nucleotides, which inhibit target gene expression post-transcriptionally by directly binding to the 3′ untranslated region (UTR) of target mRNA, which results in mRNA degradation and translation inhibition [23, 24]. The similar phenotype of miR-150 deficiency in B1a cell expansion drove us to study the possible function of miRNAs in Mysm1 deficient cells [22]. Several miRNAs have been reported to be involved in B1a cell development, including miR- 150 [22, 25], miR-146a [26], miR-17/92 [27], miR-155 [28], miR-181a [29, 30], and miR-34a [31]. To identify potential miRNAs that are involved in MYSM1-induced B1a abnormality, we performed Quantitative Reverse Transcription PCR (qRT-PCR) assays to analyze the expression level of these miRNAs in B1a cells. We found that only miR-150 was significantly and consistently decreased in both spleen (*P* = 0.0095) and peritoneal cavity (*P* = 0.0112) B1a cells from MYSM1^−/−^ mice compared with those from WT mice (Figure 2A). Meanwhile, the level of miR-150 was not altered in B1b cells from MYSM1^−/−^ mice indicating a specific role for MYSM1 in miR-150 expression in B1a cells (see Supplementary Figure S2A). To validate whether miR-150 expression is regulated by MYSM1, the expression of MYSM1 was recovered in the splenic B1a cells isolated from MYSM1^−/−^ mice by LV-MYSM1. We observed that the level of miR-150 was significantly increased after the forced expression of MYSM1 in MYSM1^−/−^ B1a cells (Figure 2B, *P* = 0.0095). Meanwhile, B1a cells from WT mice were infected with lentivirus encoding MYSM1-specific shRNA (sh-MYSM1). We found that the level of miR-150 was significantly decreased with the attenuation of MYSM1 (Figure 2B), which was similar to the results observed in B1a cells from MYSM1^−/−^ mice (Figure 2A). The percentage of B1a cells in the spleens and peritoneal cavities of miR-150^−/−^ mice is increased [22], which was similar with the phenomena observed in MYSM1^−/−^ mice.

We next sought to test the effect of miR-150 on the phenotype alteration of B1a cells. To do so, the level of miR-150 in B1a from MYSM1^−/−^ mice was recovered using miR-150 expressing-lentivirus (LV-miR-150) (see Supplementary Figure S2B). After 72 h infection of LV-miR-150, the level of surface IgM on B1a cells decreased in an inverse correlation to the increase in miR-150 (Figure 2C). This suggests that miR-150 is involved in IgM enhancement induced by MYSM1 deficiency (Figure 1C–1E).

As MYSM1 may recruit transcription factors to regulate the transcription of target genes, we tested whether MYSM1 stimulated transcription of the miR-150 gene. Genes encoding microRNA are first transcribed as large primary transcripts (pri-miRNAs). Our investigation showed that levels of pri-miR-150 were significantly reduced in B1a cells from MYSM1^−/−^ mice (Figure 2D), which suggests that the transcription of miR-150 was decreased in B1a cells with a MYSM1 deletion. To further investigate, we performed a ChIP (Chromatin Immunoprecipitation) assay in B cells from WT mice to detect the direct interaction between MYSM1 and the promoter of miR-150. The miR-150 gene is located at chromosome 19 (50004042–50004125). We designed 15 specific primer pairs of 3000 nucleotide (nt) upstream of miR-150 gene (see Supplementary Figure S2C). We found that the region approximately 1200–1000 nt upstream of the miR-150 promoter was specifically captured by anti-MYSM1-IgG (Figure 2E), which was inseparably linked with MYSM1 function [16]. Moreover, this specific interaction was not observed in MYSM1^−/−^ mice (Figure 2F).

It has been reported that transcription factor c-Myc stimulates the transcription of miR-150 [32]. c-Myc was predicted to bind to the 1200–1000 nt upstream region of the miR-150 gene by TFSEARCH (ver.1.3). We performed ChIP assay to detect the binding region of c-Myc at the promoter of miR-150. Results showed that the c-Myc antibody specifically captured the same region of the miR-150 gene promoter as the anti-MYSM1-IgG in B cells from WT mice (Figure 2G). Likewise, there was no observed interaction between c-Myc and the promoter of miR-150 gene in B cells from MYSM1^−/−^ mice (Figure 2G). In addition, we found that the level of c-Myc was not altered in B cells from MYSM1^−/−^ mice compared with WT mice as analyzed by qRT-PCR (see Supplementary Figure S2D). These results suggest that c-Myc stimulates the transcription of miR-150 in the presence of MYSM1. To further verify that MYSM1 is necessary for c-Myc binding to the promoter of miR-150, we first obtained the products captured by the antibody of MYSM1 then added the c-Myc antibody. We found that c-Myc specifically bound to the promoter of miR- 150 in the captured products of anti-MYSM1-IgG (Figure 2H). Meanwhile, co-Immunoprecipitation (co-IP) was performed to detect the interaction between MYSM1 and c-Myc and results showed that MYSM1 protein co-immunoprecipitated with c-Myc in murine RAW264.7 cells (Figure 2I).

For further investigation, the 1200–1 nt upstream region of the miR-150 promoter and its corresponding mutant (with a deletion of 1200–1000nt) were cloned into pGL6 to generate luciferase reporter vectors (pGL- miR- 150p and pGL-miR-150pm) (see Supplementary Figure S2E). These two vectors were transfected into 293T cells individually and the cells were concurrently infected with c-Myc expression lentivirus. Results showed that the deletion of the 1200– 1000 nt upstream region of miR-150 attenuated the luciferase activities of the miR- 150-promoter reporter (∼26-fold decrease compared with WT reporter pGL-miR-150p; *P* < 0.01) (Figure 2J). In addition, over-expression of c-Myc increased the luciferase activities of pGL-miR-150p, but had no effect on pGL-miR-150pm (Figure 2J).

Next we confirmed the regulatory effect of c-Myc on the transcription of miR-150 in B1a cells. Splenic B1a cells were isolated from WT mice and an inhibitor of c-Myc (10058F4) was added to the cell culture. We found that the level of pri-miR-150 in B1a was decreased (Figure 2K), while the inhibitor of c-Myc had no effect on the level of MYSM1 (Figure 2K). Next, splenic B1a cells from MYSM1^−/−^ mice were isolated and infected with LV-MYSM1 and the c-Myc inhibitor (10058F4) was added. Results showed that the inhibitor of c-Myc blocked the elevation of miR-150 that was induced by restoring MYSM1 (Figure 2L). These results suggest that MYSM1, together with c-Myc, stimulates the transcription of pri-miR-150.

### miR-150 inhibits FLT3 in B1a cells

**Figure 3:**
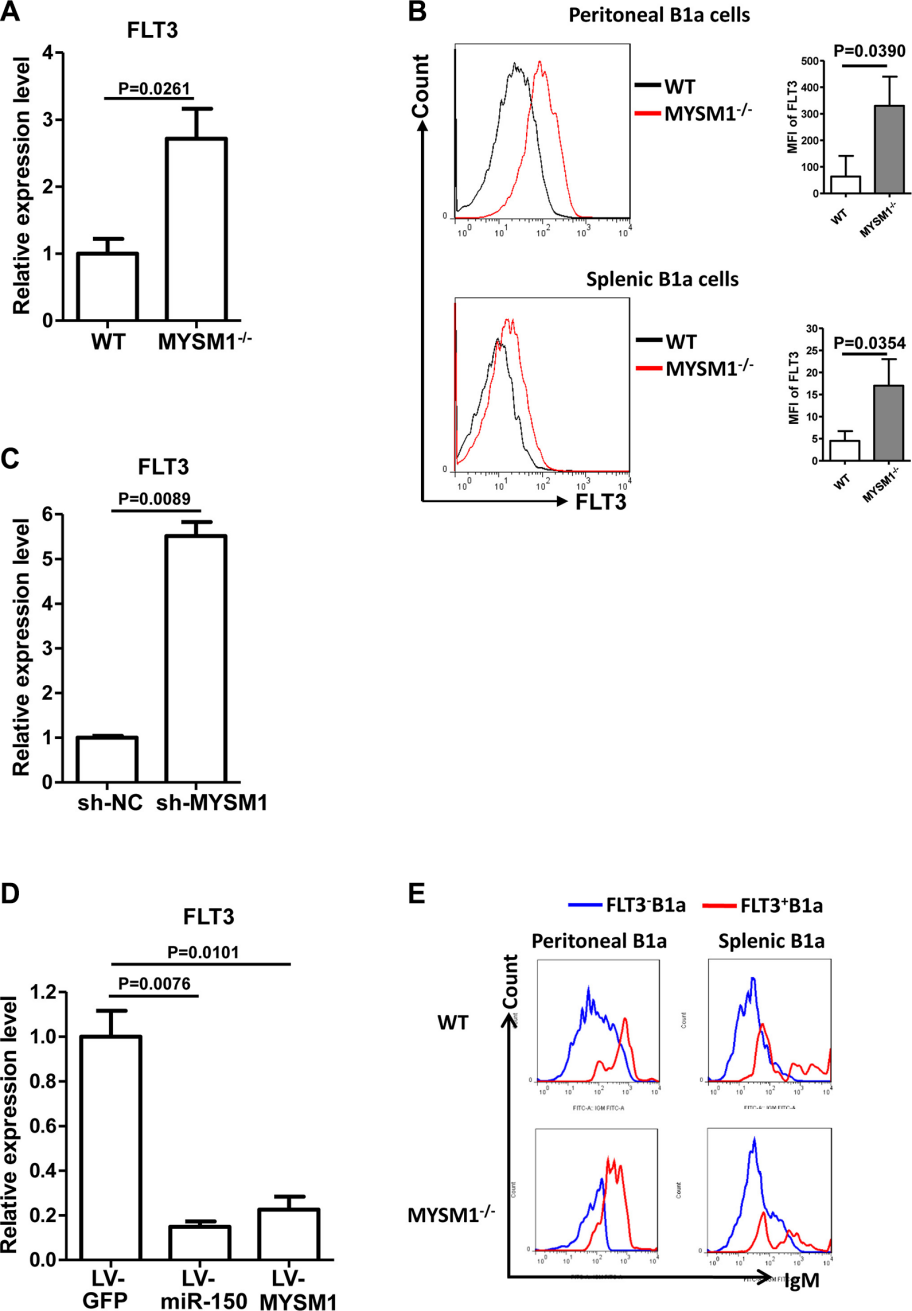
miR-150 inhibits FLT3 in B1a cells (**A**) The expression of FLT3 is increased in splenic B1a cells from MYSM1^−/−^ mice. (**B**) The expression level of FLT3 protein in B1a from MYSM1^−/−^ mice is increased compared with wild type mice. Representative flow cytometry profiles (left) and MFI (right) of FLT3 expression status on B1a cells from peritoneal cavities (top) and spleens (bottom) from homozygous MYSM1^−/−^ mice and WT littermates. *n* = 8. (**C**) FLT3 expression increases when MYSM1 is down-regulated by sh-MYSM1. (**D**) Ectopic expression of both miR-150 and MYSM1 in MYSM1^−/−^ B1a reduces the level of FLT3. B1a cells were isolated from MYSM1^−/−^ mice and transduced with the lentivirus containing the miR-150 gene (LV-miR-150) or the MYSM1 gene (LV-MYSM1). qRT-PCR results show that the increase of miR-150 or MYSM1 in MYSM1^−/−^ B1a cells represses the expression of FLT3. (a, b, c, d) Data are representative of three independent experiments and shown as the mean ± SD. Significant differences between groups were evaluated using a two-tailed Student's *t* test. (**E**) The level of surface IgM is higher in FLT3^+^ B1a cells. IgM expression status on FLT3^+^ B1a cells and FLT3^−^ B1a cells from peritoneal cavities and spleens from homozygous MYSM1^−/−^ mice and WT littermates. Data are shown from one of three repeated experiments.

MicroRNAs act by directly regulating the transcripts of target genes [24, 33]. Several targets of miR-150 have been identified, including c-myb [22, 32], notch3 [34], and FLT3 (CD135) [32]. To determine the targets that contribute to the abnormality of MYSM1^−/−^ B1a cells, the transcripts of these targets in B1a cells from MYSM1^−/−^ mice was detected by qRT-PCR. Of the targets examined, only the expression of FLT3 was increased in B1a cells from MYSM1^−/−^ mice (Figure 3A, Supplementary Figure S3).

The FMS-like tyrosine kinase 3 (FLT3) gene encodes for a membrane-bound Class III receptor tyrosine kinase (RTK) that has a critical role in normal hematopoiesis [35]. To further validate the elevation of FLT3 in MYSM1^−/−^ mice, we analyzed the protein level of FLT3 using FACS. It was found that the protein level of FLT3 in B1a from MYSM1^−/−^ mice was significantly increased compared with WT mice (Figure 3B), which was consistent with the qRT-PCR results for FLT3. Additionally, when the expression of MYSM1 was knocked down by sh-MYSM1 in the B1a cells of WT mice, the level of FLT3 increased (Figure 3C). We then demonstrated that ectopic expression of both miR-150 and MYSM1 in MYSM1^−/−^ B1a cells reduced the level of FLT3 (Figure 3D).

Upon further investigation, we found that the level of surface IgM was increased in FLT3^+^ B1a cells (Figure 3E), suggesting that the elevation of FLT3 might be related to the abnormalities of B1a cells from MYSM1^−/−^ mice.

### The percentage of FLT3^+^ B1 cells in peripheral blood is significantly elevated in SLE patients

**Figure 4:**
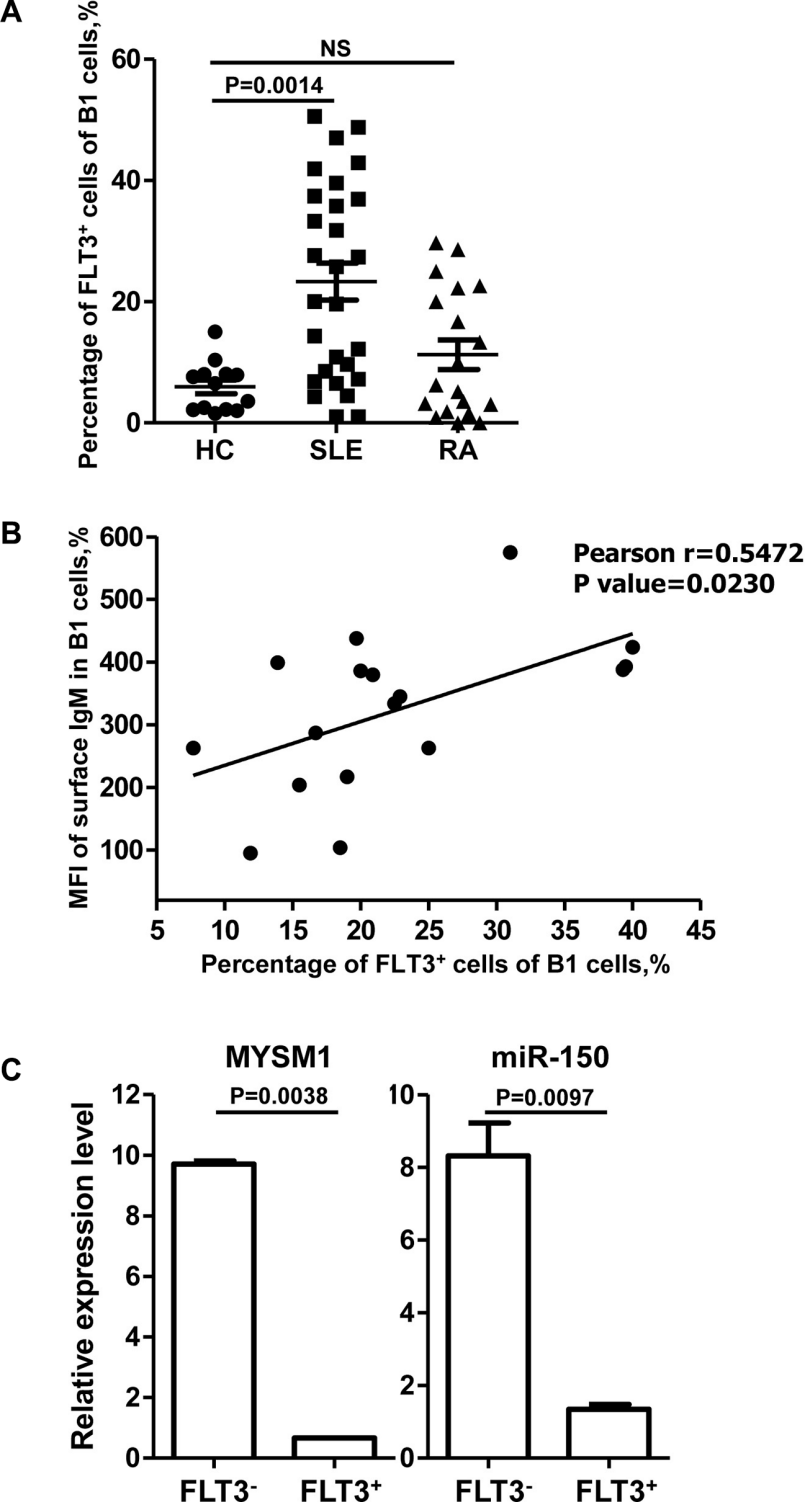
The percentage of FLT3^+^ B1 cells in peripheral blood is significantly higher in SLE patients (**A**) The percentage of FLT3^+^ B1 cells is significantly higher in SLE patients (*n* = 28) compared with healthy controls (*n* = 13). Data are shown as the mean ± SD. Significant differences between groups were evaluated using a two-tailed Student's *t* test. (NS: Not Significant). (**B**) The level of surface IgM is positively correlated with the percentage of FLT3 on B1 cells (*n* = 17). Relationship between between groups was evaluated using a two-tailed Pearson correlation coefficients. (**C**) Total RNA was extracted separately from FLT3+ and FLT3- B cells which were isolated from PBMC of 6 SLE patients by FACS. Then the levels of MYSM1 and miR-150 were measured by using qRT-PCR. The level of MYSM1 and miR-150 is lower in FLT3^+^ B cells than that in FLT3^−^ B cells. Data are representative of three independent experiments and shown as the mean ± SD. Significant differences between groups were evaluated using a two-tailed Student's *t* test.

It is now evident that B cells contribute to the pathobiology of rheumatoid arthritis (RA) and systemic lupus erythematosus (SLE) [36]. However, there are few reports about B1 cells in such diseases. Therefore we sought to detect the percentage of B1 cells in human peripheral blood in RA and SLE using the specific markers CD20^+^CD5^+^ [36]. Interestingly, we found that the percentage of B1 cells was not altered in RA and SLE compared with healthy controls (see Supplementary Figure S4). However, the percentage of FLT3^+^ B1 cells was significantly higher in SLE patients compared with healthy controls (Figure 4A). In addition, the level of surface IgM was positively correlated with the percentage of FLT3^+^ B1 cells (Figure 4B), which was consistent with the phenotype of FLT3^+^ B1a in mice.

Given that FLT3 is the target of miR-150, and MYSM1 stimulates the transcription of miR-150 in mice, we next analyzed the level of MYSM1 and miR-150 in both FLT3 positive and negative cells in humans. RNA isolated from peripheral blood of patients with SLE analyzed with qRT-PCR revealed that the level of MYSM1 and miR-150 was lower in FLT3^+^ B cells than in FLT3^−^ B cells (Figure 4C) suggesting that FLT3 elevation was due to a decrease in MYSM1.

## DISCUSSION

Here, we have identified a pathway comprised of MYSM1/miR-150/FLT3, which inhibits B1a cell proliferation. We further demonstrate that MYSM1 recruits c-Myc and stimulates the transcription of miR-150. Then the expression of FLT3, the target of miR-150, is inhibited. MYSM1 knockout in mice leads to a higher percentage of B1a cells *in vivo*. Moreover, we also revealed that the percentage of FLT3^+^ B1 cells is significantly elevated in peripheral blood from patients with SLE and the level of MYSM1 is decreased in FLT3^+^ B1 cells from these patients, which suggests that this pathway is altered in SLE patients.

Several studies have reported the biological function of MYSM1 [15–21]. MYSM1 deubiquitinates monoubiquitinated histone H2A and directs the recruitment of transcription factors to the promoter of target genes, including EBF1 and PU.1. Here, we show that MYSM1 and c-Myc bind to the same region (approximately 1200–1000 nt upstream) of the promoter of miR-150 in B1a cells from WT mice but c-Myc cannot bind to the promoter in MYSM1^−/−^ mice. These results suggest that MYSM1 recruits transcription factor c-Myc to the promoter of miR-150, thereby positively regulating its transcription.

In miR-150-deficient mice, splenic B1a cells and peritoneal B1a cells are increased [22]. In our study, the level of miR-150 in MYSM1^−/−^ mice is significantly lower, and the percentage of splenic and peritoneal B1a cells is increased, which is in accordance with the phenotype observed in miR-150-deficient mice. C-Myb is another target of miR-150 and is thought to be involved in B cell development [22]. However, our results show that the level of c-Myb is not altered in B1a cells from MYSM1^−/−^ mice compared with WT, whereas the level of FLT3 is negatively correlated with miR-150 in B1a cells.

FLT3 is a membrane-bound receptor tyrosine kinase (RTK) which is involved in the proliferation, differentiation, and apoptosis of hematopoietic cells [37]. The human FLT3 gene has 85% amino acid sequence homology with mouse FLT3. Here, we found that some B1 cells in human and in mouse can express FLT3. Moreover, the percentage of FLT3-positive B1 cells is higher in patients with SLE compared to healthy controls. However, whether or not this subgroup of B1 cells contributes to the pathogenesis of SLE needs to be addressed in the future.

In conclusion, we reveal a novel pathway involving MYSM1, miR-150, and FLT3, which inhibits B1a cell proliferation and a defect in this pathway may contribute to the pathogenesis of SLE.

## MATERIALS AND METHODS

### Study approval

All experimental animal protocols for this study are in accordance with the national guidelines for the use of animals in scientific research. Additional approval was granted by the Animal Care and Use Committee of Beijing Institute of Basic Medical Sciences, with the approval number BMS-12021103.

### Mice

MYSM1-deficient mice were generated as described previously [16]. In summary, they were generated by crossing MYSM1 mRNA truncation-first floxed mice (MYSM1 tm1a/tm1a). In all experiments, WT littermates (^+/+^) matched by gender and age were used for controls. Mice were maintained in a pathogen-free barrier facility, and all experiments were performed in accordance with the Institute of Basic Medical Sciences Guide for Laboratory Animals.

### Patients characteristics

All patients were recruited consecutively from the Department of Rheumatology and Immunology, Peking University People's Hospital. Patients with infections were carefully excluded on clinical grounds. Healthy blood donors with no history of autoimmune diseases or treatment with immunosuppressive agents served as controls. This study was approved by the ethics committee of the Peking University People's Hospital. After oral and written informed consent had been obtained, whole blood samples (10 ml) were taken from all participants (after acclimatization at room temperature for 1 hour).

### Antibodies and flow cytometry

Sample preparation, cytometric analysis, and cell sorting were performed as described previously [16]. Single-cell suspensions of spleens and peritoneal cavities were prepared and red blood cells were lysed using BD Pharm Lyse (BD Biosciences). Cells were first stained for 20 min at 4°C with CD16/32 Fc-blocking antibody (2.4G2) in flow cytometry buffer, followed by incubation with a “cocktail” of antibodies conjugated to fluorescein isothiocyanate (FITC), phycoerythrin (PE), peridinine chlorophyll protein (PerCP) or allophycocyanin (APC). The following antibodies from BD Biosciences or eBioscience were used for flow cytometry: anti-B220 (RA3–6B2), anti-CD19 (1D3), anti-CD43 (S7), anti-CD135/FLT3 (A2F10.1), anti-CD5 (53–7.3), and anti-IgM (R6–60.2). Data were collected on a FACSCanto II (BD) and analyzed with FlowJo software (TreeStar). For B cell sorting, cells from spleens and peritoneal cavities were first stained with a cocktail of antibodies then sorted by flow cytometry. Cell populations were isolated by FACSAria as follows: from peritoneal cavity: B1a cells (CD19^+^B220^lo^CD5^+^CD43^+^), B1b cells (CD19^+^B220^lo^CD5^−^CD43^+^), and B2 cells (B220^+^CD19^+^); from spleen: B1a cells (CD19^+^B220^lo^CD5^+^CD43^+^) and B2 cells (CD19^+^B220^lo^CD5^−^CD43^+^).

### Enzyme-linked immunospot (Elispot) assay

Splenic and peritoneal B1a cells were sorted by FACSAria cell sorter. IgM Production capacity of B1a cells was examined based on the instructions for Elispot^plus^ for Mouse IgM (Mabtech). Briefly, B1a cells were resuspended in RPMI-1640 with 10% FCS and stimulated with LPS (100 ng/ml, Sigma) on plates coated with anti-mouse IgM antibody. Cells were incubated overnight at 37°C with 5% CO_2_ and 95% humidity. After washing, plates were treated with anti-IgM-biotin secondary antibody for 2 hours and then streptavidin conjugated-alkaline phosphatase for 1 hour at room temperature. The blue spots were developed with 5-bromo-4-chloro-3-indolyl phosphate/NBT substrate. Enumeration of the colored spots on the dried Elispot plates was performed using the automated AID Elispot reader (Autoimmun Diagnostika GmbH, Strasbourg, Germany). The percentage of IgM-secreting B1a cells was calculated by dividing the number of positive spots by the total number of cells seeded in each well.

### Lentivirus production and transduction

Recombinant lentiviral vectors (sh-MYSM1 and sh-NC; LV-MYSM1 and LV-GFP) were produced and transduced as described in our previous publications [16, 17]. Recombinant lentiviral vectors containing miR- 150 were purchased from Genechem (Shanghai, China).

### Chromatin immunoprecipitation

Chromatin was immunoprecipitated according to the manufacturer's instructions (Cell Signaling). Briefly, cell suspensions were crosslinked with 1% (vol/vol) formaldehyde. Chromatin was isolated, digested by mung bean nuclease (MNase), sheared by sonication, and immunoprecipitated with antibodies. Immunoprecipitated DNA was washed and eluted according to the manufacturer's instructions. Eluted DNA and sheared input material was analyzed by real time PCR. For sequential-ChIP experiments, complexes from initial anti-MYSM1 ChIP were eluted, diluted, and then reimmunoprecipitated with c-Myc antibody.

### RNA extraction and cDNA production

Total RNA was extracted from cells using Trizol (Invitrogen) according to the manufacturer's instructions. For cDNA synthesis, 1 μg of RNA was mixed with 500 ng of olig (dT) (Promega) or microRNA specific primers (invitrogen). The RNA-primer mixture was incubated at 65°C for 5 min, followed by a snap freeze in ice bath for 2 min. Then samples were incubated at 42°C for 45 min with 5 μl of 5×first-strand buffer, 2 μl of 5 mM dNTP, 20 U of RNasin (Takara), 1 μl of M-MLV reverse transcriptase (Promega), and distilled water to a total volume of 25 μl. The reverse transcriptase was inactivated at 70°C for 10 min and then chilled on ice.

### Quantitative reverse transcription PCR (qRT- PCR)

The qPCR reaction mixture contained 12.5 μl of 2×qPCR mix (Fermentas), 0.3 μM of gene-specific forward and reverse primers, and 1 μl of template, made up to a final volume of 25 μl with distilled water. Cycling parameters were set as follows: initial activation step at 95°C for 10 min, denaturation at 95°C for 30 s, annealing at 58°C for 30 s, and extension at 72°C for 20 s. Melting curve analysis was performed between 58°C and 95°C with stepwise fluorescence acquisition at every 1°C s^–1^. Melting curves observed for each gene were confirmed to correspond to the correct amplicon size by agarose or polyacrylamide gel electrophoresis of the PCR products. The levels of gene expression were calculated by relative quantification using GAPDH or U6 snRNA as the endogenous reference genes. All samples were amplified in triplicate and the data analysis was carried out using the MxPro qPCR system software (Stratagene). The ΔCt value was calculated by subtracting the Ct value for GAPDH or U6 snRNA from the Ct value for the gene of interest.

### Vector construction and luciferase reporter assay

To generate a luciferase reporter construct, the promoter and mutant promoter of pre-miR-150 were inserted upstream of firefly luciferase in pGL6. Cells were transfected with luciferase reporters, using pRL-TK as control vector. Luciferase activity was measured using the Dual-Luciferase Assay kit (Promega) with a beta-counter luminometer. Relative luciferase activity was calculated as ratio of the raw firefly luciferase activity and the renilla luciferase activity.

### Western blotting

Cells were lysed in lysis buffer (50 mM Tris (pH 7.4), 150 mM NaCl, 1% Triton X-100, 1 mM sodium orthovanadate, 1% sodium deoxycholate, 0.1% SDS, 1 uM leupeptin). The same amount of protein samples were subjected to 15% SDS-PAGE and the proteins were blotted onto Hybond-ECL nitrocellulose membrane (Amersham Biosciences). The membrane was blocked in 5% non-fat dry milk at 37°C for 1 h, probed with antibodies against target proteins for 12 h at 4°C, and washed twice in PBS with 0.5% Tween 20 (PBST). Next the membrane was incubated in a 1:5,000 solution of HRP-conjugated goat anti-rabbit or -mouse secondary antibody at room temperature for 1 h. After further washing with PBST, the membrane was assayed by the enhanced chemiluminescence (ECL) Western blotting detection system.

### Flow cytometric analyvsis of human peripheral blood sample

Whole blood samples were collected in evacuated tubes containing EDTA as the anticoagulant and processed within 4 h of drawing. Peripheral blood leukocytes were labeled using the whole blood lysis method below. 100 ul whole blood was aliquoted per test, and the red blood cells were lysed using RBC Lysis Buffer (Biolegend) and washed with phosphate buffered saline (PBS). Acquired leukocytes were resuspended in 50 uL of PBS supplemented with 10% fetal calf serum. 5 ul FcR-blocking reagent (Miltenyi Biotec) was added to each tube for 10 min to block nonspecific antibody binding. The cells were stained with anti-CD20-PE-Cy7, anti-CD5-RITC, anti-IgM-PE, and anti-FLT3/CD135-APC for 30 min at 4°C then analyzed via flow cytometry (BD).

### Statistical analysis

All experiments were repeated at least three times and the results are expressed as the mean ± SD. All quantitative data were analyzed using Student *t*-tests, unless otherwise indicated. All tests performed were two-sided. *P* < 0.05 was considered to be statistically significant. GraphPad Prism software was used for statistical analyses.

## SUPPLEMENTARY MATERIALS FIGURES


