# Supplementary material for: MYSM1/miR-150/FLT3 inhibits B1a cell proliferation
Source: Oncotarget. 2016 Aug 31;7(42):68086–96. doi: 10.18632/oncotarget.11738 (PMC5356540; doi:10.18632/oncotarget.11738)
Supplement: Supplementary file 1 [file oncotarget-07-68086-s001.pdf]

MYSM1/miR-150/FLT3 inhibits B1a cell proliferation

Supplementary Materials

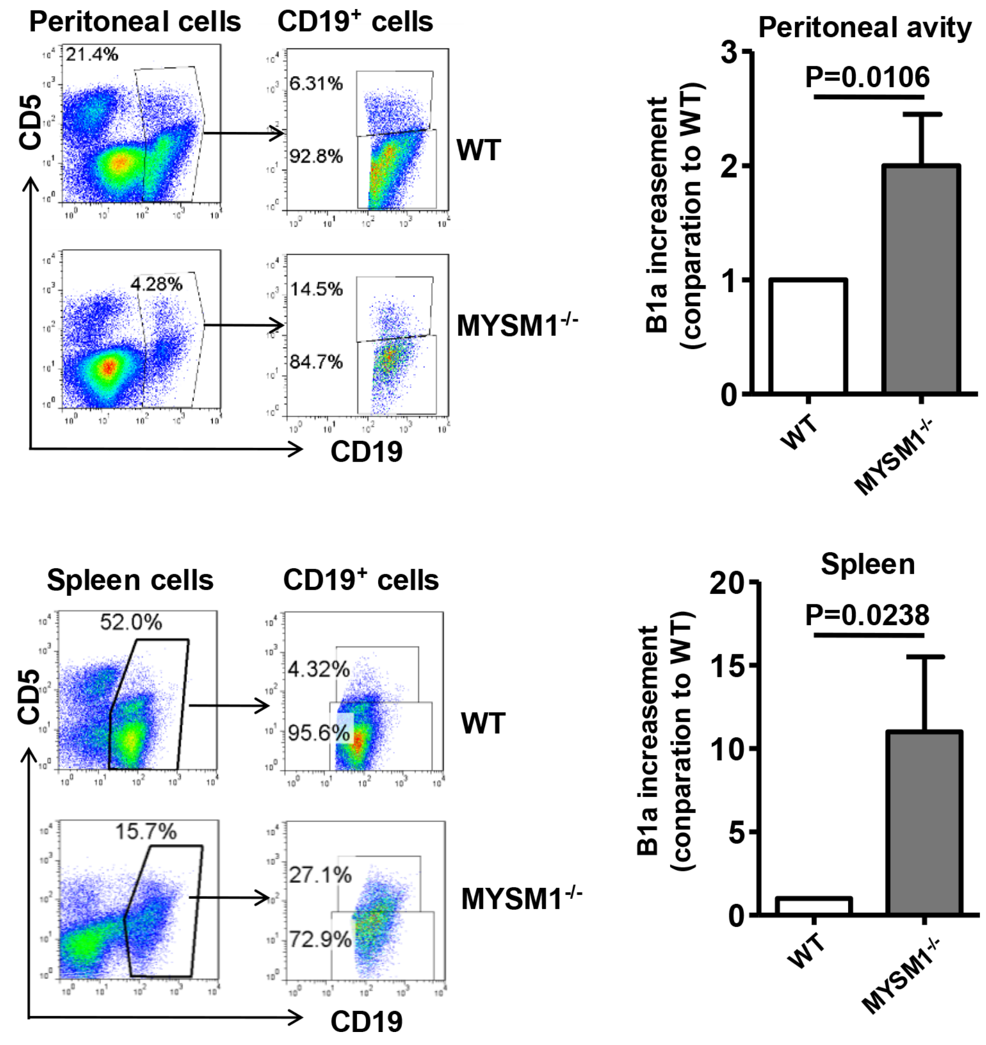

Supplementary Figure S1: Increased B1a cell frequency in MYSM1-deficient mice. Representative flow cytometry profiles (left) and increased fold of B1a cells (right) in the peritoneal cavity (top) and spleen (bottom) from homozygous MYSM1$^{-/-}$ mice and WT littermates designated by CD19^{+}CD5^{+}. Data are representative of three independent experiments and shown as the mean ± SD. Significant differences between groups were evaluated using a two-tailed Student's *t* test.

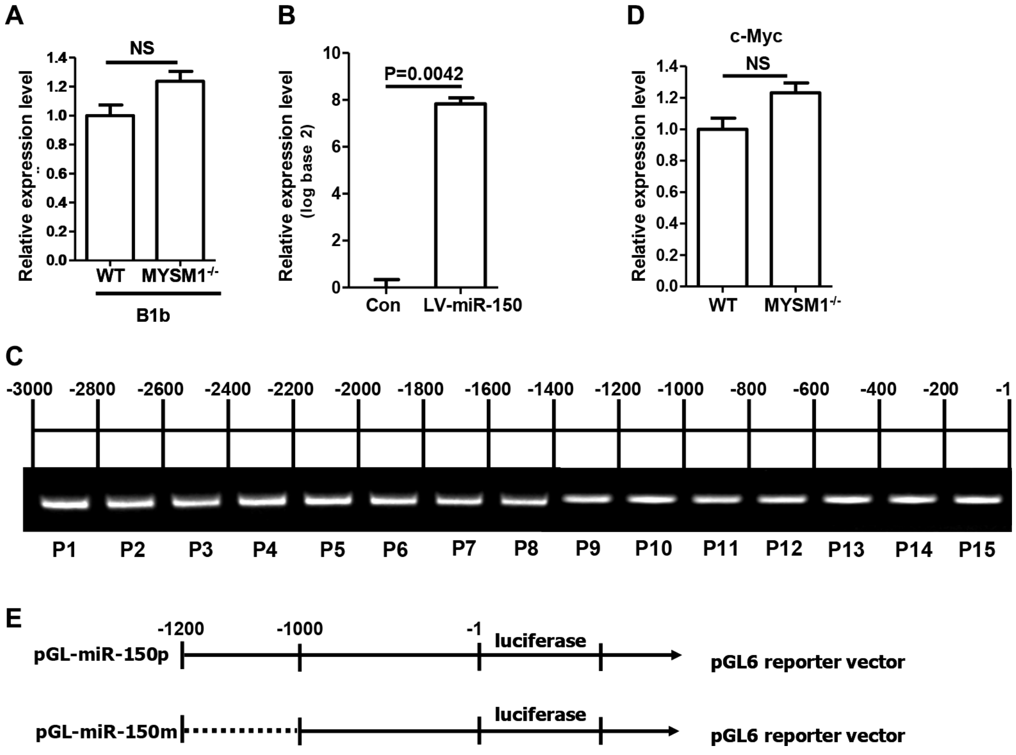

Supplementary Figure S2: MYSM1 regulates the transcription of miR-150 in B-1a cells with c-Myc. (**A**) The level of miR-150 is not altered in B-1b cells from MYSM1$^{-/-}$ mice. (**B**) The level of miR-150 in B1a from MYSM1$^{-/-}$ mice is increased using a miR-150 expressing-lentivirus (LV-miR-150). Data are representative of three independent experiments and shown as the mean ± SD. Significant differences between groups were evaluated using a two-tailed Student's *t* test. (**C**) 15 specific primer pairs specific for detection of the 3000 nt upstream of miR-150 gene. Data are representative of three independent experiments and shown as the mean ± SD. Significant differences between groups were evaluated using a two-tailed Student's *t* test. (**D**) The level of c-myc is not altered in B cells from MYSM1$^{-/-}$ mice compared with wild type mice. (**E**) The region of -1200~-1000 nt of miR-150 promoter and its corresponding mutant were cloned into the pGL6 to generate the luciferase reporter vectors.

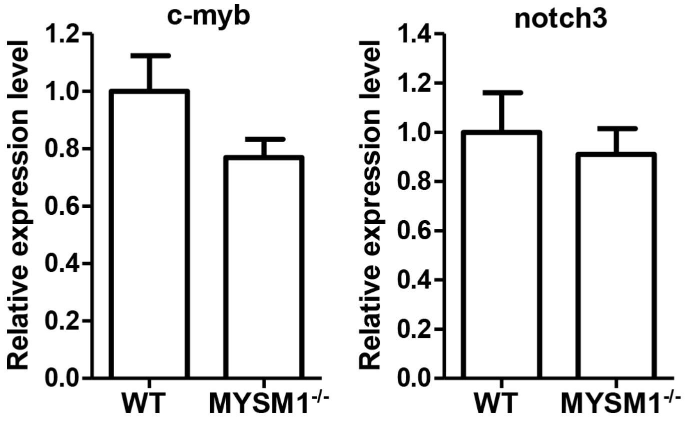

Supplementary Figure S3: There is no significant change in other targets of miR-150 in MYSM1$^{-/-}$ mice compared with wild type mice.

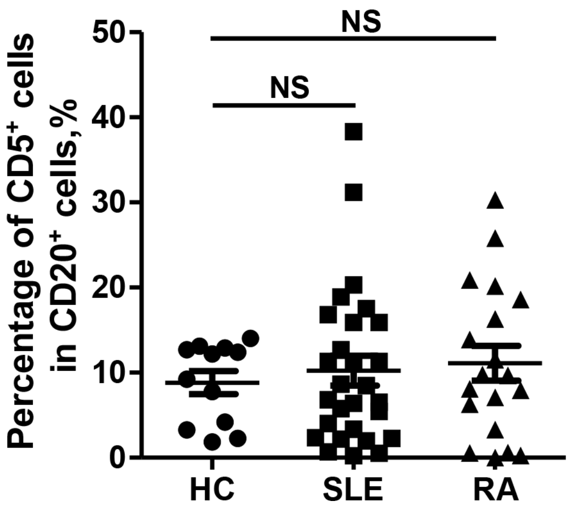

Supplementary Figure S4: The percentage of B1 cells is not altered in RA and SLE compared with healthy controls.
